# Editorial: Feeding and Nutritional Strategies to Reduce Livestock Greenhouse Gas Emissions

**DOI:** 10.3389/fvets.2021.717426

**Published:** 2021-07-02

**Authors:** Paula Toro-Mujica, Manuel González-Ronquillo

**Affiliations:** ^1^Instituto de Ciencias Agroalimentarias, Animales y Ambientales, Universidad de O'Higgins, San Fernando, Chile; ^2^Facultad de Medicina Veterinaria y Zootecnia, Universidad Autónoma del Estado de México, Toluca, Mexico

**Keywords:** ruminant, tropical forage, secondary metabolites, simulation model, enteric methane mitigation

Ruminants play a fundamental role in the world's food and the sustainability of agricultural production systems (1). The nutritional composition of their products ensures the supply of essential nutrients, including amino acids, fatty acids, vitamins, and minerals. Furthermore, the proper management of productive systems favors the conservation of the landscape and allows carbon sequestration by improving the health of the soil through the increase of organic matter (2). However, the current rate of production to which animal production systems are subjected tends to exceed their natural productive capacity. This makes it necessary to include many agricultural and veterinary inputs, breaking the production/carbon sequestration balance and polluting air, water, and soil (3). In this scenario, livestock production has been strongly questioned, among other reasons, due to the emission of greenhouse gases. Estimates indicate that greenhouse gas (GHG) production from livestock represents 14.5% (7.1 Gt CO_2eq_ annual^−1^) of total anthropogenic emissions, with enteric emissions contributing 35% of the sector's emissions (4). The reduction of GHG emissions and the adaptation to the new climate change scenarios are urgent needs in the agriculture and forestry sector. Animal feeding is one of the variables with the most significant influence on total GHG production and production efficiency (5). The latter is expressed either through traditional indicators such as the conversion rate and weight gain or through the carbon footprint. Thus, the search for comprehensive strategies to mitigate GHG emissions from livestock systems must incorporate strategies associated with animal feed and nutrition and ultimately evaluate their effect through productive parameters.

In this Research Topic, seven articles analyze and propose feed and nutritional strategies aimed at reducing GHG emissions. The studies presented by Enriquez et al., Gaviria-Uribe et al., and Su and Chen analyze the effect of incorporating forage and shrub species into ruminant's diets. Enriquez et al. study the effect of incorporating a fresh mixed annual ryegrass and berseem clover forage into the winter diet of dairy cows in intensive production systems that base feeding on a total mixed ration with a base of conserved summer crops. Gaviria-Uribe et al. evaluate the effect of the nutritional composition and voluntary intake of diets based on tropical forages upon CH_4_ emissions from zebu steers. The tropical forages used by Gaviria-Uribe et al. include novel Cayman grass and Leucaena shrub legumes. Through a review article, Su and Chen summarize the effects of the inclusion of Moringa oleífera leaf in broiler chickens, layers, pigs, aquatic animals, and ruminants. *Moringa oleifera* is commonly used for its protein content; however, its inclusion has also been related to a decrease in methane production. Therefore, the incorporation of leaves, flour, seeds, or extracts of *Moringa oleifera*, thanks to the natural capacity of the species to inhibit methanogenesis, has reported successful results in research carried out in buffalos (6), goats (7), beef cattle (8), and dairy cows (9). As Su and Chen point out, *Moringa oleifera* has a high concentration of secondary metabolites, which would regulate fermentation conditions and associations between microorganisms. This characteristic has also been observed in *Leucaena leucocephala* species in which high contents of condensed tannins are found (10).

Regarding the use in animal feeding of species with high concentrations of secondary metabolites, recent research has evaluated the incorporation of seaweed into ruminant diets with results, in some cases, promising (11, 12). Through his Perspective article, Vijn et al. consider the use of seaweed and provides valuable recommendations for the design of research associated with the use of seaweed as an enteric methane mitigant in the stages of seaweed production, animal feed production, and livestock production. Ku-Vera et al. perform an exhaustive review on the role of secondary plant metabolites on enteric methane mitigation, explaining the possible mechanisms of action of tannins, saponins, essential oils, flavonoids on enteric CH_4_ mitigation. In addition to secondary metabolites, there is a wide range of additives that have been evaluated in ruminant diets to mitigate GHG, including lipids, ionophores, nitrogen sources, and biocarbon (13–16). In the case of lipids, these are offered mainly through oil-rich seeds, such as cottonseed (17), linseed (13, 18), and canola oil (16, 19). Among ionophores, one of the most used is monensin, whose incorporation in ruminant diets has been related to increased feed conversion efficiency. The efficiency increase will be produced by decreasing gram-positive bacteria and protozoa (20). Regarding nitrogen sources in sheep (21) and cattle (22), there are examples of how the incorporation of urea or nitrates help to reduce methane emissions. Studies on the effects on methane emissions of the inclusion of biocarbon in ruminant diets are still scarce, due to this, Terry et al. analyze the effect of the inclusion of the pine enhanced biochar (EB) in heifer diets on various parameters associated with ruminal fermentation, including methane (CH_4_) emissions. Finally, Toro-Mujica, using the results of previous research about the effect of the use of additives in the production of GHG, raises the need for evaluation at the livestock system level. With this objective, using a simulation model, Toro-Mujica determines the effect of modifying management practices and incorporating two additives (monensin and canola oil) into the diet on bio-economic variables and carbon footprint of cow-calf systems.

Considering the current climate change scenario, this Research Topic contributes to the knowledge of feed and nutritional strategies for the mitigation of livestock greenhouse gas emissions, and their evaluation at the production system level.

## Author Contributions

PT-M wrote the first draft. MG-R wrote and edited the second draft. All authors contributed to the article and approved the submitted version.

## Conflict of Interest

The authors declare that the research was conducted in the absence of any commercial or financial relationships that could be construed as a potential conflict of interest.
